# Emergency Medicine Reimagined: Development of the Patient Rights Index for Emergency Medicine

**DOI:** 10.5811/westjem.62375

**Published:** 2026-10-14

**Authors:** Matthew Ryan

**Affiliations:** University of Florida, College of Medicine, Department of Emergency Medicine, Gainesville, Florida

## Abstract

**Introduction:**

Emergency medicine has evolved beyond episodic acute care to encompass a growing range of clinical, social, and system-level responsibilities. This scholarly perspective proposes the Patient Rights Index in Emergency Medicine (PRI-ED), a conceptual framework designed to organize recurring domains through which emergency departments promote access to care, patient safety, relief of suffering, autonomy, and dignity.

**Methods:**

The PRI-ED was developed through iterative domain generation informed by clinical observation, professional experience, and targeted review of peer-reviewed literature, policy analyses, and professional organizational reports. No primary data collection, systematic review, formal consensus process, or empirical validation was performed.

**Results:**

We identified 12 domains that represent recurring clinical, operational, and system-level challenges encountered in emergency medicine. These domains were organized within a standardized framework linking domain-specific objectives, intervention strategies, measurable indicators, and relevant policy or funding mechanisms.

**Conclusion:**

The PRI-ED is a proposed conceptual framework for organizing and evaluating domains in which emergency medicine intersects with access, safety, autonomy, relief of suffering, and dignity. The framework is intended to inform future implementation, evaluation, and policy discussion and requires feasibility testing and outcome validation before effectiveness can be determined.

## INTRODUCTION

Emergency medicine (EM) has evolved beyond its traditional role as a specialty of episodic acute care. Since its formal recognition as a discipline defined by immediacy, stabilization, and universal access, emergency departments (ED) have become a major point of entry into the healthcare system for patients experiencing acute illness, psychiatric crises, substance use disorders, homelessness, barriers to healthcare access, and end-of-life needs. More than 155 million visits occur annually in United States EDs, reflecting a role that extends beyond acute medical stabilization.^1^–^4^

The Emergency Medical Treatment and Labor Act (EMTALA) has played a central role in this evolution by establishing a critical safety net within the US healthcare system. However, while EMTALA ensures access to emergency care, it does not address healthcare fragmentation, underinsurance, continuity gaps, administrative barriers, and persistent inequities in access to comprehensive care.^5^–^11^ Thus, EDs function at the intersection of acute illness, social vulnerability, and healthcare access.^12^

This reality presents both challenges and opportunities for the specialty. Emergency physicians remain resuscitationists, dedicated to rapid diagnosis, stabilization, and lifesaving interventions, while also serving as essentialists who respond to the consequences of fragmented healthcare systems and unmet social, behavioral, and public health needs. In this capacity, EM routinely encounters issues of access, safety, autonomy, relief of suffering, and dignity. Within this framework, dignity refers to preserving patient autonomy while ensuring respectful, safe, equitable, and compassionate care.

Although many of these challenges have been studied individually, including psychiatric boarding, homelessness, violence, palliative care, patient safety, healthcare access, and EMTALA reform, they are often examined within their respective clinical, operational, or policy domains.^13^ To our knowledge, no prior framework has attempted to organize these interconnected responsibilities within a unified model for EM.

This scholarly perspective proposes the Patient Rights Index in Emergency Medicine (PRI-ED), a conceptual framework that identifies and organizes twelve recurring clinical, operational, and system-level domains through which EDs promote access to care, patient safety, autonomy, relief of suffering, and dignity. By linking these domains to operational strategies, measurement indicators, and policy mechanisms, the PRI-ED is intended to inform future implementation, evaluation, advocacy, and systems-level improvement efforts within EM. The framework is intended to illustrate measurable areas for evaluation and intervention rather than provide an exhaustive inventory of challenges facing the specialty.

The domains are summarized in Table 1. The framework is intended to identify measurable objectives and inform future implementation, evaluation, and policy discussion. Although grounded in the US healthcare system and EMTALA framework, many underlying concepts may have broader relevance to emergency care systems internationally.

## METHODS

### Framework Development

This paper presents a scholarly perspective proposing the PRI-ED, a conceptual framework organizing recurring domains in which EM intersects with patient access, safety, autonomy, relief of suffering, and dignity. The framework was developed through targeted review of peer-reviewed literature, policy analyses, and organizational reports, supplemented by iterative refinement informed by clinical experience and observation. No primary data collection, systematic review, formal consensus process, or empirical validation was performed.

### Domain Identification and Selection

Initial domains were generated from recurring clinical, operational, and systems-level challenges encountered in contemporary EM practice and refined through review of EM, health policy, public health, patient safety, behavioral health, palliative care, and social EM literature. We included domains if they substantially influenced patient access, safety, autonomy, relief of suffering, or dignity and were potentially amenable to operational, educational, policy, funding, or quality-improvement interventions. Domains with substantial conceptual overlap were consolidated to improve clarity and usability. The final framework consists of 12 domains.

### Framework Construction

For each domain, we developed a standardized PRI-ED table consisting of an objective, scope, intervention strategies, measurable indicators, and relevant policy or funding mechanisms. These elements were selected to connect conceptual priorities with practical implementation and future evaluation.

### Methodological Considerations

The PRI-ED is intended as a conceptual framework rather than an empirical model and should be viewed as hypothesis-generating. Domain selection reflects the author’s synthesis of recurring challenges in EM informed by clinical experience and targeted literature review and is not intended to represent an exhaustive inventory of issues facing the specialty. The literature review informing framework development was targeted rather than systematic and was primarily grounded in the US healthcare system and EMTALA environment.

## RESULTS

The final PRI-ED framework consists of 12 domains affecting ED practice (Table 1). Each domain is organized into a standardized PRI-ED table containing five elements: objective, scope, intervention strategies, measurement indicators, and funding/policy mechanisms (Tables 2–13). Collectively, these 12 PRI-ED tables provide a common structure for translating conceptual priorities into measurable implementation strategies.

## DISCUSSION

The following sections describe the 12 PRI-ED domains and their associated tables. The accompanying PRI-ED tables illustrate how these concepts may be translated into measurable objectives, intervention strategies, and potential policy or funding mechanisms within EM practice.

### Psychiatric Emergency Care

Emergency departments have increasingly become the de facto entry point for patients in psychiatric crisis.^14^ Nationwide, there are only 28.4 inpatient psychiatric beds per 100,000 people—far below the benchmark of 60 per 100,000.^15^ Mental health-related ED visits among youth increased 56% between 2011 and 2020, and nearly one in five US adults lives with a mental health condition.^16^,^17^ Psychiatric emergencies now represent a substantial share of ED care. Funding remains fragmented. Medicaid is the largest payor, and parity enforcement remains inconsistent.^18^ Together with limited inpatient capacity and shortages of community behavioral health resources, these factors contribute to prolonged psychiatric boarding, with patients awaiting placement or discharged without sustainable follow-up.^19^

The ED can respond by embedding crisis stabilization units,^14^ redesigning behavioral health-safe rooms, and expanding telepsychiatry, particularly in under-resourced settings. Integration of psychiatry, social work, and peer navigators enables warm handoffs, while staff training in trauma-informed care and de-escalation supports dignity and safety. At the policy level, expansion of crisis infrastructure (including the 988 Suicide & Crisis Lifeline) and efforts to improve parity implementation may help strengthen access to behavioral health services.^18^ Yet hallway placement, restraints, and lack of privacy continue to compound delays and may hinder patient-centered recovery efforts.^19^ Table 2 outlines operational strategies to reduce psychiatric emergency care.

### End-of-Life and Palliative Care in the Emergency Department

Emergency departments are seeing more patients with serious, life-limiting illnesses. Traditionally focused on acute stabilization, the specialty is often asked to initiate values-based care.^10^,^20^ Over 80% of US hospitals with 50 or more beds report some form of palliative program, yet access remains limited in for-profit and rural settings, contributing to disparities in access.^21^ Even where services exist, ED integration is inconsistent; patients may be discharged without goals-of-care discussions, symptom management, or connection to specialty teams.^22^ Workforce shortages and limited training compound these gaps.^23^,^24^

Emergency departments can respond by embedding palliative screening tools at triage, implementing comfort-focused protocols for pain, dyspnea, and agitation, and establishing automatic triggers for specialty consultation when appropriate.

Education remains central. Palliative care skills are increasingly recognized as important competencies for emergency physicians, yet standardized national requirements are uneven. Many programs now incorporate structured training through initiatives such as Education in Palliative and End-of-Life Care for Emergency Medicine or dedicated rotations, emphasizing primary-level communication and symptom management skills sustained through continuing education.

Federal efforts such as the Palliative Care and Hospice Education and Training Act, along with reimbursement parity, could strengthen workforce capacity and support ED-based implementation.^24^ Timely goals-of-care conversations may help align treatment with patient preferences and reduce unwanted interventions at the end of life.^22^ Table 3 outlines a framework for translating these principles into measurable practice.

### Sexual Assault Response

Sexual assault is prevalent and carries lasting consequences. In the ED, the response extends beyond treating injuries and collecting evidence to provide safety, dignity, and support. Women bear the overwhelming burden of sexual violence, while male and nonbinary survivors may face stigma that further impedes disclosure and access to care.^25^ Yet many EDs cannot guarantee timely access to Sexual Assault Nurse Examiners (SANE) or trained forensic clinicians.^25^ The result is variability in survivor-centered care and missed opportunities for timely medical, forensic, and advocacy services. There is no universally standardized approach to integrating sexual assault care into EM training, and many residency programs rely on SANE services or local resources for education.^26^ Consequently, physicians may be variably prepared to address survivors’ medical, emotional, and legal needs.^27^

These gaps extend to other forms of interpersonal violence, including domestic violence, elder abuse, and child abuse, where the ED may represent the only point of contact with the healthcare system. Timely recognition, trauma-informed care, and clear pathways to protection and advocacy remain essential.

Hospitals can support timely access to expert care through onsite SANE staffing or regional referral networks. Trauma-informed protocols addressing medical treatment, prophylaxis, forensic evidence collection, and linkage to advocacy services may be integrated into ED workflows. Physician education, partnerships with advocacy organizations, and funding mechanisms such as Violence Against Women Act programs, state victim compensation funds, and Health Resources and Services Administration (HRSA) rural health grants may help sustain these services.^28^ Table 4 illustrates how these commitments can be incorporated into everyday ED practice.

### Improved Follow-Up and Continuity of Care

Most patients seen in the ED are treated and discharged, and for many this is their only contact with the healthcare system.^29^ Discharge instructions may be difficult to understand, referrals may be delayed, and barriers such as transportation limitations, inadequate insurance coverage, or unstable housing often remain unresolved.^30^ Patients with chronic illness, medical complexity, socioeconomic disadvantage, limited health literacy, or poor access to primary care are particularly vulnerable to fragmented transitions and recurrent ED utilization. These challenges reflect the tension between vertical care, which is episodic and crisis-focused, and horizontal care, which emphasizes continuity, prevention, and coordination across healthcare settings.

Emergency medicine can help bridge this divide through strategies that strengthen continuity beyond the ED encounter. Direct handoffs to primary care, specialty services, or community-based resources can improve care coordination and reduce barriers to follow-up, particularly when supported by case management and social work services.^31^ Technology-enabled approaches may further support continuity through telehealth follow-up visits, automated reminders, and nurse-led outreach programs that reinforce discharge instructions and facilitate early recognition of clinical deterioration.^32^ In parallel, sustainable reimbursement models are needed to support these activities. Existing Centers for Medicare & Medicaid Services (CMS) programs, including Transitional Care Management and Chronic Care Management reimbursement pathways, may provide a foundation for adapting and supporting ED-initiated follow-up efforts. Table 5 outlines these concepts. In this model, “treat and release” becomes “treat and reconnect.”

### Homelessness and Multi-Visit Patients

People experiencing homelessness visit the ED two to four times more often than the general population, and unsheltered individuals are nearly twice as likely as sheltered individuals to require ED care within a year.^33^ Many encounters are associated with untreated chronic illness, substance use disorders, psychiatric crises, and lack of stable housing. Once in the ED, this population is more likely to be admitted and up to eight times more likely to be readmitted within 30 days.^33^ Without housing, discharge instructions and prescriptions may have limited impact, contributing to recurrent utilization.

Evidence suggests this cycle can be interrupted. Programs linking homeless patients to primary care, behavioral health, and case management have reduced ED visits by nearly 50% within a year.^34^ Housing First initiatives have reduced ED use in multiple cities while lowering Medicaid expenditures.^35^,^36^ Medical respite programs provide safe recovery environments after discharge and are expanding through CMS demonstration projects.

Emergency departments can contribute by embedding social workers, care coordinators, and community partners into care pathways. Dedicated teams for high utilizers may develop individualized care plans linking patients to sobering centers, detoxification programs, psychiatric stabilization facilities, or supportive housing.^33^,^36^ Street medicine teams and mobile crisis units extend these efforts beyond hospital walls.

Multi-visit patients present a related challenge. Many cycle through the ED because of insurance lapses, benefits confusion, poor care coordination, or barriers to follow-up.^36^ Tele-insurance navigation and tele-social work may help connect patients with coverage specialists, housing referrals, medication assistance, and transportation resources, potentially reducing avoidable return visits.^32^

States are increasingly using Medicaid waivers to support tenancy assistance, case management, and housing-related services. California’s CalAIM and Project Homekey initiatives illustrate the impact of aligning health financing with housing solutions.^36^ In Central Florida, homelessness costs an estimated $31,000 per person annually, nearly triple the cost of permanent supportive housing.^37^ These findings underscore the close relationship between housing stability and health. Table 6 highlights strategies to interrupt recurrent ED use and strengthen connections to housing, behavioral health, and community-based services.

### Social Work and Case Management

Many ED visits are influenced by unmet social needs such as food insecurity, unstable employment, insurance gaps, unsafe housing, or exposure to violence.^38^,^39^ Rural and critical-access hospitals may lack dedicated social workers entirely, relying on nurses or physicians to assemble referrals that address social concerns. Without real-time case management, patients may leave with medical issues addressed but underlying crises unresolved, contributing to repeat visits and preventable deterioration.^40^–^42^

Emergency departments can strengthen care by embedding social workers and care navigators into clinical workflows and linking patients to community resources, either in person or through telehealth-enabled models that extend support to smaller or rural EDs.^36^,^37^ Early involvement of case managers improves throughput, facilitates safe discharges, and reduces unnecessary admissions.^40^ Social workers and community health workers can connect patients to insurance enrollment, transportation, medication assistance, housing support, and domestic violence resources, improving continuity beyond the ED.^42^

Table 7 outlines operational strategies, measurement elements, and potential policy mechanisms to expand social work and case management capacity, including CMS Innovation Models, HRSA integration grants, and state Medicaid waivers.

### Global Health Applied Locally

Global health has long emphasized efficiency, equity, and resourcefulness in low-resource settings. These principles provide a useful framework for addressing inequities and resource constraints. Yet underserved communities in the US, both urban and rural, often face barriers to care that resemble challenges encountered in resource-limited settings.^43^ Emergency medicine can adapt lessons from global health to local practice. Low-cost innovations, task-shifting, rapid triage models, and community outreach have proven effective abroad and can be reimagined here.^44^,^45^ Partnerships with local clinics, shelters, schools, and faith-based organizations can mirror the networks built in global health. Training emergency physicians in both global and community health may facilitate adaptation of successful strategies across diverse practice settings.^46^

Mobile outreach programs, often staffed by residents, students, and emergency physicians, already demonstrate what is possible. In some communities, these programs have expanded access to preventive services, health screening, vaccination efforts, and injury-prevention initiatives.^46^ Federal and state funding streams (HRSA, Centers for Disease Control and Prevention, Substance Abuse and Mental Health Services Administration, Medicaid waivers) may help sustain these efforts.^47^

Global health is not only a mission abroad but also a framework for improving care within our own communities. Table 8 highlights strategies to translate global health principles into local EM practice.

### Sustaining Emergency Access

Access to emergency care is a defining responsibility of EM and a critical component of the healthcare safety net.^11^ Since 1986, EMTALA has mandated emergency care regardless of ability to pay. As an unfunded mandate, EMTALA has contributed to substantial uncompensated care, creating ongoing financial challenges for hospitals and health systems.^48^ Rural hospitals have closed under these pressures, while others remain bound by EMTALA yet lack meaningful access for patients.^49^ Interpretation and application of EMTALA requirements may vary across institutions, contributing to challenges such as psychiatric boarding, specialty coverage shortages, and crowding.^49^ In reproductive emergencies, clinicians may also face conflicts between EMTALA’s federal stabilization requirements and state abortion laws when maternal health is threatened.^50^

Although EMTALA is part of the EM core curriculum, understanding of the law varies widely. Emergency physicians are generally more familiar with the provisions, but specialists and trainees may lack clarity regarding responsibilities and liabilities, leaving clinicians vulnerable to error and patients vulnerable to delay.^48^

Emergency medicine can reaffirm EMTALA as a cornerstone of emergency access. Modernization efforts may include financial support for uncompensated care, updated guidance addressing psychiatric boarding and specialty access, and educational initiatives that promote consistent understanding and application of the law. Greater regulatory clarity may also help define EMTALA obligations when emergency stabilization requirements intersect with other state or federal policies affecting patient care. Table 9 outlines operational strategies and policy mechanisms that may strengthen implementation of EMTALA’s core access-to-care principles.

### Workplace Safety and Violence Prevention

Workplace violence remains a significant and growing concern in EM.^50^ Nearly half of emergency physicians report having been physically assaulted at work, and more than 70% of emergency nurses experienced verbal or physical abuse in the prior year.^51^,^52^ Healthcare workers are five times more likely to experience workplace violence than workers in other industries, with EDs representing one of the highest-risk settings.^53^ The consequences extend beyond staff well-being and may affect the care environment. Psychiatric boarding, crowding, prolonged lengths of stay, and resource constraints can contribute to tension among patients, families, and staff. In addition, variability in security resources, de-escalation training, incident reporting, and postevent support may limit institutional efforts to address workplace violence effectively.^52^,^53^

Safety may be viewed as a core quality measure within emergency care. Violence-prevention programs may include standardized de-escalation training, environmental safety measures, incident-reporting systems, and postevent support services. Federal standards and legislative initiatives such as the Workplace Violence Prevention for Health Care and Social Service Workers Act may further strengthen these efforts. Table 10 highlights strategies to build safer environments for patients, families, and staff.

### Balanced Performance Measurement

Performance measurement plays an essential role in EM, informing quality improvement, operational management, accountability, and resource allocation. Traditional metrics such as throughput, length of stay, and productivity provide valuable information about system performance, but they may not fully capture other dimensions of care that are important to patients, clinicians, and healthcare organizations.^54^ Growing interest has emerged in measuring aspects of care that extend beyond operational efficiency, including patient experience, communication, care transitions, and clinician well-being.^55^ These domains may provide a more comprehensive understanding of ED performance and complement existing measures of quality and productivity.

Measurement strategies that incorporate both operational and patient-centered outcomes may help organizations identify opportunities to improve care while supporting a sustainable clinical environment.^56^ Examples of additional measures that could be considered include patient understanding of discharge instructions, successful linkage to follow-up care, patient-reported experiences of respect and communication, and other indicators that reflect the quality of interactions and care transitions.^57^ Such measures are intended to complement rather than replace traditional operational and clinical metrics.

The PRI-ED framework proposes a broader approach to performance assessment that incorporates both technical and human dimensions of care. Table 11 summarizes potential strategies for expanding measurement frameworks to better reflect the diverse goals of emergency medicine.

### Ethical Use of Artificial Intelligence and Technology

Artificial intelligence (AI) is being integrated into emergency care, with applications ranging from clinical documentation and decision support to predictive analytics, telehealth, and operational management.^58^ In many settings, AI tools are assisting clinicians in identifying patients at risk for sepsis, forecasting resource utilization, drafting clinical notes, and supporting triage and discharge decisions.^58^ These technologies have the potential to improve efficiency, enhance decision-making, and reduce administrative burden, allowing clinicians to devote more time to direct patient care.

At the same time, the integration of AI into EM raises important ethical and operational considerations. Algorithmic performance depends on the quality and representativeness of training data, creating the potential for bias and unintended disparities in care.^59^ Performance may also vary across patient populations and clinical settings, underscoring the importance of ongoing evaluation and validation. Experiences with widely deployed tools, including sepsis prediction models, have highlighted the challenges of balancing sensitivity, specificity, and clinical usability in real-world practice.^60^

As AI adoption accelerates, EM will require governance frameworks that promote transparency, accountability, and patient-centered implementation. Algorithms should be evaluated for potential bias, monitored for unintended consequences, and deployed with appropriate clinician oversight.^61^ Rather than replacing clinical judgment, AI should function as a tool that supports decision-making while preserving the vital role of the patient-clinician relationship.^62^ Priority should be given to technologies that improve communication, education, safety, and care coordination while reducing administrative burden.

The PRI-ED framework recognizes that technological innovation should be evaluated not only by its operational impact but by its effects on safety, equity, patient experience, and clinician well-being. Table 12 summarizes strategies to support the responsible integration of AI and emerging technologies into emergency care.

### Professional Identity and Values Alignment

Emergency medicine operates within increasingly complex clinical, operational, financial, and regulatory environments. As healthcare systems evolve, maintaining alignment between professional values and organizational priorities remains an important challenge.^62^ Core principles including patient-centered care, universal access, ethical practice, and service to vulnerable populations continue to shape the identity of the specialty.^63^,^64^

Professional identity is influenced both by training, mentorship, and clinical experience, and by the priorities embedded within healthcare systems. Performance metrics, incentive structures, and institutional goals signal what organizations value and can influence how clinicians define success in practice. Measures related to throughput, productivity, and efficiency are important components of emergency care but may not fully capture other dimensions of practice that many clinicians view as central to the specialty’s mission.^64^,^65^

The PRI-ED framework recognizes that evaluation measures help shape professional culture. By incorporating patient experience, dignity, communication, equity, safety, and clinician well-being alongside traditional operational and clinical metrics, PRI-ED seeks to align accountability systems with the broader commitments that have historically defined emergency medicine.^63^,^65^ Rather than replacing existing measures, the framework provides a more balanced assessment of performance that reflects both the technical and human dimensions of care.

Residency education, mentorship, faculty development, and professional organizations remain important mechanisms for reinforcing these values. Aligning educational priorities, organizational incentives, and performance measures may help clinicians meet operational demands while remaining grounded in the foundational principles of the specialty.^64^ Table 13 summarizes the PRI-ED framework for supporting professional identity and values alignment through educational, organizational, and policy-based strategies.

## LIMITATIONS

This paper presents a conceptual framework and scholarly perspective rather than an empirical research study. The PRI-ED was developed through iterative domain generation informed by clinical observation, professional experience, targeted literature review, policy analyses, and organizational reports. No systematic review, formal consensus methodology (eg, Delphi process), external stakeholder review, psychometric testing, or prospective validation was performed. Accordingly, domain selection and organization reflect the author’s interpretation of recurring challenges in EM and may be influenced by professional experience, practice environment, and selection bias. Other reasonable frameworks, domains, or organizational structures could be proposed.

The framework is intended to organize concepts and stimulate discussion rather than establish standards of care, quality benchmarks, accreditation requirements, or regulatory expectations. Although individual domains draw upon published literature and existing policy initiatives, the PRI-ED framework has not been validated, and its effects on patient outcomes, operational performance, equity, or healthcare utilization remain unknown.

Implementation may vary substantially across institutions because of differences in resources, workforce capacity, local partnerships, reimbursement structures, regulatory environments, and community needs. In addition, the framework was developed within the US healthcare system and may not be fully generalizable to international settings with different financing models, regulatory structures, workforce configurations, cultural expectations, and social service infrastructures. Future work should evaluate the feasibility, reliability, and practical utility of PRI-ED through stakeholder engagement, implementation studies, and formal validation efforts.

## CONCLUSION

The Patient Rights Index in Emergency Medicine is a proposed conceptual framework for organizing recurring domains in which emergency medicine intersects with access, safety, autonomy, relief of suffering, and dignity. By linking these domains to potential operational, educational, quality improvement, and policy interventions, PRI-ED provides a structured approach for future discussion and evaluation. Further work is needed to assess the feasibility, reliability, and impact of framework components across diverse emergency care settings.

## Figures and Tables

**Table 1:** The twelve domains of the Patient Rights Index in Emergency Medicine framework.

Domain No.	Focus Area	Central Theme
1	Psychiatric Emergency Care	Improving access, safety, and continuity for patients with behavioral health needs
2	End-of-Life and Palliative Care	Aligning emergency care with patient goals and preferences
3	Sexual Assault Management	Ensuring timely access to trauma-informed forensic services
4	Follow-Up and Continuity	Strengthening care transitions and postdischarge follow-up
5	Homelessness and Multi-visit Patients	Addressing social determinants that contribute to recurrent emergency care use
6	Social Work and Case Management	Integrating social and care coordination resources into emergency care
7	Global Health Applied Locally	Applying health equity and population health principles in local practice
8	Sustaining Emergency Access	Adapting emergency access policies to contemporary healthcare systems
9	Workplace Safety and Violence Prevention	Promoting a safe environment for patients, families, and healthcare workers
10	Balanced Performance Measurement	Broadening performance assessment beyond operational metrics
11	Ethical Use of AI and Technology	Supporting responsible and equitable implementation of emerging technologies
12	Professional Identity and Values Alignment	Aligning professional values with organizational priorities

*AI*, artificial intelligence.

**Table 2:** Patient Rights Index in Emergency Medicine (PRI-ED) domain: psychiatric emergency care. The PRI-ED domain within a conceptual framework proposing operational strategies, measurement elements, and policy mechanisms to address psychiatric emergency care and boarding within emergency departments in a scholarly perspective describing the PRI-ED framework for emergency medicine.

Component	Description
Objective	Improve timeliness, safety, and dignity of care for patients presenting with acute psychiatric or behavioral health crises.
Scope	ED patients presenting with psychiatric or behavioral emergencies requiring stabilization, inpatient admission, crisis services, or structured community follow-up.
Intervention Strategies	Develop or partner with CSUs linked to EDs. Design or retrofit behavioral-health–safe treatment spaces to reduce restraint use. Expand access to real-time telepsychiatry consultation, particularly in rural or under-resourced settings. Establish escalation pathways that notify hospital leadership when boarding thresholds are exceeded.
Measurement Elements	Time from ED arrival to psychiatric placement or crisis disposition. Total psychiatric boarding hours per admission. Use of restraints or involuntary medication events. Stratified data by race, ethnicity, payor, sex, and language to identify disparities.
Funding and Policy Mechanisms	Medicaid Section 1115 waivers and demonstration projects. SAMHSA crisis services block grants. State behavioral-health parity enforcement initiatives. Regional crisis-care integration funding streams.

*CSU*, crisis stabilization unit; *ED*, emergency department; *SAMHSA*, Substance Abuse and Mental Health Services Administration.

**Table 3:** Patient Rights Index in Emergency Medicine (PRI-ED) domain: end-of-life and palliative care. The PRI-ED domain within a conceptual framework proposing operational strategies, measurement elements, and policy mechanisms to integrate palliative and end-of-life care within emergency departments in a scholarly perspective describing the PRI-ED framework for emergency medicine.

Component	Description
Objective	Improve alignment of emergency care with patient goals, reduce non-beneficial interventions, and enhance goal-concordant care and quality of life for patients with serious illness.
Scope	ED patients with advanced chronic illness, life-limiting conditions, serious frailty, or repeated high-acuity presentations who may benefit from goals-of-care clarification or palliative consultation.
Intervention Strategies	Implement standardized goals-of-care screening triggers in triage or admission workflows. Develop rapid-access palliative consultation pathways (in-person or telehealth). Embed code-status verification protocols within ED intake processes. Provide clinician education on primary palliative communication skills.
Measurement Elements	Proportion of eligible patients with documented goals-of-care discussion. ED-initiated palliative consult rates. ICU admissions among patients with documented limitations of life-sustaining treatment. Successful linkage to palliative services following ED discharge.
Funding/Policy Mechanisms	CMS reimbursement for advance-care planning discussions. Value-based purchasing programs tied to serious-illness care metrics. Institutional quality incentives for documentation of code status. Accountable care organization shared-savings programs.

*CMS*, Centers for Medicare & Medicaid Services; *ED*, emergency department; *ICU*, intensive care unit.

**Table 4:** Patient Rights Index in Emergency Medicine (PRI-ED) domain: sexual assault response. The PRI-ED domain within a conceptual framework proposing operational strategies, measurement elements, and policy mechanisms to support survivor-centered sexual assault care within emergency departments in a scholarly perspective describing the PRI-ED framework for emergency medicine.

Component	Description
Objective	Provide timely, trauma-informed medical and forensic care that prioritizes safety, consent, and dignity for survivors of sexual assault.
Scope	All patients presenting to the ED following sexual assault, regardless of sex, age, or identity.
Intervention Strategies	Maintain 24/7 access to trained SANE or teleSANE services. Embed trauma-informed documentation templates within the electronic health record. Establish standardized referral pathways to advocacy and crisis-support services at initial clinical contact. Provide clinician education in survivor-centered communication and medical-legal responsibilities. Formalize partnerships with community advocacy and legal-response organizations.
Measurement Elements	Proportion of shifts with SANE or teleSANE availability. Time from ED arrival to forensic examination when indicated. Percentage of survivors connected to advocacy services within 72 hours. Patient-reported measures of perceived safety, respect, and informed consent during care.
Funding/Policy Mechanisms	VAWA and OVC grants. HRSA rural outreach and telehealth funding programs. State and local crime victim compensation funds.

*ED*, emergency department; *HRSA*, Health Resources and Services Administration; *OVC*, Office for Victims of Crime; *SANE*, Sexual Assault Nurse Examiner; *VAWA*, Violence Against Women Act.

**Table 5:** Patient Rights Index in Emergency Medicine (PRI-ED) domain: follow-up and continuity of care. The PRI-ED domain within a conceptual framework proposing operational strategies, measurement elements, and policy mechanisms to strengthen post–emergency department continuity of care in a scholarly perspective describing the PRI-ED framework for emergency medicine.

Component	Description
Objective	Improve safe transitions from ED care to outpatient, specialty, or community-based follow-up.
Scope	ED patients discharged with new diagnoses, medication changes, unresolved diagnostic uncertainty, or identified social risk factors requiring structured follow-up.
Intervention Strategies	Implement real-time scheduling of primary or specialty follow-up prior to discharge. Develop ED-based care navigation or transition coordinators. Integrate automated follow-up communication (text, phone, portal). Establish high-risk discharge lists for structured 48–72 hour callback review.
Measurement Elements	Percentage of discharged patients with documented follow-up appointment scheduled. 7- and 30-day ED revisit rates. Successful contact rates for high-risk discharge callbacks. Stratified revisit data by payer, race, ethnicity, and sex.
Funding/Policy Mechanisms	TCM billing codes. Value-based reimbursement programs tied to readmission or utilization metrics. Medicaid managed-care care-coordination incentives. Institutional shared-savings arrangements with outpatient partners.

*ED*, emergency department; *TCM*, transitional care management.

**Table 6:** Patient Rights Index in Emergency Medicine (PRI-ED) domain: homelessness and multi-visit patients. The PRI-ED domain within a conceptual framework proposing operational strategies and measurable approaches to improve stabilization, housing linkage, and continuity for patients experiencing homelessness in a scholarly perspective describing the PRI-ED framework for emergency medicine.

Component	Description
Objective	Strengthen ED-to-housing pathways to reduce recurrent utilization and improve stability.
Scope	Patients with chronic homelessness, unstable housing, or frequent ED use without established primary or behavioral health care.
Intervention Strategies	Integrate social work and case management early in the ED visit. Develop individualized stabilization and discharge plans. Establish referral pathways to medical respite, temporary shelter, and Housing First programs. Coordinate with supportive housing, behavioral health, and community outreach teams. Assist with identification, benefits enrollment, and transportation access.
Measurement Elements	Reduction in repeat ED visits among enrolled patients. Rates of successful housing or primary care linkage. Trends in ED and hospital utilization among frequent users.
Funding/Policy Mechanisms	Medicaid 1115 waiver programs. CMS innovation or value-based care demonstrations. State or local housing-health initiatives. Philanthropic or community development grants.

*CMS*, Centers for Medicare & Medicaid Services; *ED*, emergency department.

**Table 7:** Patient Rights Index in Emergency Medicine (PRI-ED) domain: social work and case management. The PRI-ED domain within a conceptual framework proposing operational strategies, measurement elements, and policy mechanisms to strengthen social and care coordination services within emergency departments in a scholarly perspective describing the PRI-ED framework for emergency medicine.

Component	Description
Objective	Improve structured linkage to social services and community resources at the time of ED discharge.
Scope	ED encounters involving identified housing instability, safety concerns, transportation barriers, medication access challenges, or behavioral health needs.
Intervention Strategies	Ensure real-time access to social work services (in-person or tele-social work). Integrate community health workers or care navigators into ED discharge workflows. Develop standardized referral pathways to shelters, food programs, transportation, and FQHCs. Implement brief multidisciplinary discharge huddles for complex cases.
Measurement Elements	Percentage of eligible encounters with documented social work involvement. ED revisit rates among high social-risk patients. Documented successful referral completion within 7–14 days.
Funding/Policy Mechanisms	CMS care coordination reimbursement pathways. HRSA community health integration grants. SAMHSA behavioral health block grants. Medicaid managed-care coordination incentives.

*CMS*, Centers for Medicare & Medicaid Services; *ED*, emergency department; *FQHC*, federally qualified health center; *HRSA*, Health Resources and Services Administration; *SAMHSA*, Substance Abuse and Mental Health Services Administration.

**Table 8:** Patient Rights Index in Emergency Medicine (PRI-ED) domain: global health applied locally. The PRI-ED domain within a conceptual framework proposing operational strategies and measurable approaches to strengthen local community partnerships and outreach in a scholarly perspective describing the PRI-ED framework for emergency medicine.

Component	Description
Objective	Strengthen ED-community partnerships to improve access and continuity for underserved populations.
Scope	Community clinics, shelters, schools, public health agencies, and ED-affiliated faculty, residents, and learners.
Intervention Strategies	Establish an ED-community advisory group to identify shared priorities. Develop mobile or community-based outreach clinics aligned with local need. Integrate outreach participation into faculty and resident service credit systems. Formalize referral pathways between ED and community partners.
Measurement Elements	Number of active community partnerships. Outreach event participation counts. Proportion of outreach participants successfully linked to follow-up care.
Funding/Policy Mechanisms	HRSA community outreach funding. Local public health grants. Institutional service-learning support structures.

*ED*, emergency department; *HRSA*, Health Resources and Services Administration.

**Table 9:** Patient Rights Index in Emergency Medicine (PRI-ED) domain: sustaining emergency access. The PRI-ED domain within a conceptual framework proposing strategies to support consistent, patient-centered EMTALA application within emergency departments in a scholarly perspective describing the PRI-ED framework for emergency medicine.

Component	Description
Objective	Promote consistent understanding and operationalization of EMTALA requirements across emergency care systems.
Scope	EDs, hospital leadership, residency training programs, and specialty transfer networks.
Intervention Strategies	Establish an interdisciplinary EMTALA review group. Implement structured EMTALA education modules for clinicians and trainees. Standardize stabilization and transfer protocols across high-risk domains (psychiatric, obstetric, specialty). Conduct periodic case-based review sessions.
Measurement Elements	Completion rates of EMTALA training modules. Frequency of protocol review updates. Variability in transfer documentation compliance.
Funding/Policy Mechanisms	Institutional compliance resources. CMS regulatory alignment initiatives. Quality-improvement funding streams.

*CMS*, Centers for Medicare & Medicaid Services; *ED*, emergency department; *EMTALA*, Emergency Medical Treatment and Labor Act.

**Table 10:** Patient Rights Index in Emergency Medicine (PRI-ED) domain: workplace safety and violence prevention. The PRI-ED domain within a conceptual framework proposing structured approaches to improve safety for patients and staff within emergency departments in a scholarly perspective describing the PRI-ED framework for emergency medicine.

Component	Description
Objective	Reduce workplace violence and improve preparedness for safety incidents within the ED.
Scope	All clinicians, staff, learners, and patients within the ED environment.
Intervention Strategies	Implement routine de-escalation training. Conduct simulation-based safety drills. Standardize incident reporting pathways. Review environmental safeguards (secure access, panic systems). Provide structured postincident debriefing and support.
Measurement Elements	Staff participation rates in safety training. Incident reporting rates and review completion. Staff perception-of-safety survey results.
Funding/Policy Mechanisms	OSHA workplace safety initiatives. Institutional safety budgets. State violence-prevention grants.

*ED*, emergency department; *OSHA*, Occupational Safety and Health Administration.

**Table 11:** Patient Rights Index in Emergency Medicine (PRI-ED) domain: balanced performance measurement. The PRI-ED domain within a conceptual framework proposing strategies to integrate patient-centered measures into emergency department quality systems in a scholarly perspective describing the PRI-ED framework for emergency medicine.

Component	Description
Objective	Incorporate dignity-centered and patient-experience measures into routine ED quality reporting.
Scope	Departmental quality dashboards, operational reporting systems, and patient feedback processes.
Intervention Strategies	Convene a multidisciplinary metrics review group. Pilot 1 or 2 dignity-linked indicators alongside throughput metrics. Implement brief patient micro-surveys (SMS or electronic).
Measurement Elements	Number of dignity-focused metrics adopted. Trends in patient-reported respect and understanding scores.
Funding/Policy Mechanisms	Value-based purchasing programs. CMS patient-experience reporting frameworks.

*CMS*, Centers for Medicare & Medicaid Services; *ED*, emergency department; *PRI-ED*, Patient Rights Index in Emergency Medicine; *SMS*, short message service.

**Table 12:** Patient Rights Index in Emergency Medicine (PRI-ED) domain: artificial intelligence (AI) and technology governance. The PRI-ED domain within a conceptual framework proposing guardrails for ethical AI and technology integration within emergency departments in a scholarly perspective describing the PRI-ED framework for emergency medicine.

Component	Description
Objective	Ensure transparent, ethical, and bias-aware implementation of AI tools in ED care and education.
Scope	AI-driven triage, documentation, and clinical decision-support systems used in emergency care.
Intervention Strategies	Develop departmental AI governance policies. Conduct bias and performance reviews prior to implementation. Provide staff training on AI limitations and oversight. Integrate periodic re-evaluation of AI performance.
Measurement Elements	Completion of AI governance policy. Number of AI tools formally reviewed. Staff AI training participation rates.
Funding/Policy Mechanisms	Institutional innovation funding. Federal digital health grant programs (e.g., HRSA telehealth grants, CMMI programs, NIH digital health research funding).

*AI*, artificial intelligence; *CMMI*, CMS Innovation Center; *CMS*, Centers for Medicare & Medicaid Services; *ED*, emergency department; *HRSA*, Health Resources and Services Administration; *NIH*, National Institutes of Health.

**Table 13:** Patient Rights Index in Emergency Medicine (PRI-ED) domain: professional identity and values alignment. The PRI-ED domain within a conceptual framework proposing strategies to sustain professional identity, ethical reflection, and mission alignment within emergency medicine in a scholarly perspective describing the PRI-ED framework for emergency medicine.

Component	Description
Objective	Sustain alignment between emergency medicine practice and its core values of universal access and nonjudgmental care.
Scope	Departmental culture, residency training, and faculty development programs.
Intervention Strategies	Incorporate structured ethics or values reflection sessions into conferences. Maintain and periodically review departmental mission statements. Develop case-based discussions addressing moral complexity in emergency medicine. Establish mentorship pathways supporting professional meaning and resilience.
Measurement Elements	Number of value-focused sessions held annually. Participation rates in ethics case conferences. Staff and resident survey measures of professional alignment.
Funding/Policy Mechanisms	Institutional professional development budgets. GME funding structures.

*GME*, graduate medical education.

## References

[1] Tang N, Stein J, Hsia RY (2010). Trends and characteristics of US emergency department visits, 1997–2007. JAMA.

[2] Rosen P (1979). The biology of emergency medicine. J Am Coll Emerg Med.

[3] Centers for Disease Control and Prevention, National Center for Health Statistics Emergency department visits.

[4] Wu N, Woloski JR (2024). Emergency department versus primary care use: a patient perspective. PRiMER.

[5] Augustine J (2023). A sobering year for emergency departments and their patients.

[6] Vizient, Inc (2025). From every angle: emergency department overcrowding.

[7] Hsia RY, Brownell J, Wilson S (2013). Trends in adult emergency department visits in California by insurance status, 2005–2010. JAMA.

[8] Santo L, Peters ZJ, DeFrances CJ (2021). Emergency department visits among adults with mental health disorders: United States, 2017–2019. NCHS Data Brief.

[9] Kushel MB, Perry S, Bangsberg D (2002). Emergency department use among the homeless and marginally housed: results from a community-based study. Am J Public Health.

[10] Grudzen CR, Richardson LD, Johnson PN (2016). Emergency department–initiated palliative care in advanced cancer: a randomized clinical trial. JAMA Oncol.

[11] Zibulewsky J (2001). The Emergency Medical Treatment and Active Labor Act (EMTALA): what it is and what it means for physicians. Proc (Bayl Univ Med Cent).

[12] Beauchamp TL, Childress JF (2019). Principles of biomedical ethics.

[13] Hansen K, Boyle A, Holroyd B (2020). Updated framework on quality and safety in emergency medicine. Emerg Med J.

[14] Zeller SL, Calma NM, Stone A (2014). Effects of a dedicated regional psychiatric emergency service on boarding of psychiatric patients in area emergency departments. West J Emerg Med.

[15] Treatment Advocacy Center (2024). Psychiatric bed supply need per population.

[16] Bommersbach TJ, McKean AJ, Olfson M (2023). National trends in mental health–related emergency department visits among youth, 2011–2020. JAMA.

[17] Substance Abuse and Mental Health Services Administration (2023). Key substance use and mental health indicators in the United States: results from the 2022 National Survey on Drug Use and Health.

[18] Beronio K, Glied S, Frank RG (2014). How the Affordable Care Act and Mental Health Parity and Addiction Equity Act greatly expand coverage of behavioral health care. J Behav Health Serv Res.

[19] Nicks BA, Manthey DM (2012). The impact of psychiatric patient boarding in emergency departments. Emerg Med Int.

[20] Kistler EA, Morrison RS, Richardson LD (2015). Emergency department-triggered palliative care in advanced cancer: proof of concept. Acad Emerg Med.

[21] Dumanovsky T, Augustin R, Rogers M (2016). The growth of palliative care in U.S. hospitals: a status report. J Palliat Med.

[22] Smith AK, McCarthy E, Weber E (2012). Half of older Americans seen in emergency department in last month of life: most admitted to hospital, and many die there. Health Aff (Millwood).

[23] Lupu D (2010). Estimate of current hospice and palliative medicine physician workforce shortage. J Pain Symptom Manage.

[24] Lamba S, Pound A, Rella JG (2012). Emergency medicine resident education in palliative care: a needs assessment. J Palliat Med.

[25] Campbell R, Patterson D, Lichty LF (2005). The effectiveness of sexual assault nurse examiner (SANE) programs: a review of psychological, medical, legal, and community outcomes. Trauma Violence Abuse.

[26] American College of Emergency Physicians (2013). Evaluation and management of the sexually assaulted or sexually abused patient.

[27] Wasco SM, Campbell R, Howard A (2004). A statewide evaluation of services provided to sexual assault survivors. J Interpers Violence.

[28] Health Resources and Services Administration (2024). Rural health care services outreach program.

[29] Rui P, Kang K, Ashman JJ (2016). National hospital ambulatory medical care survey: 2016 emergency department summary tables. https://www.cdc.gov/nchs/data/ahcd/nhamcs_emergency/2016_ed_web_tables.pdf.

[30] Soril LJ, Leggett LE, Lorenzetti DL (2015). Reducing frequent visits to the emergency department: a systematic review of interventions. PLoS One.

[31] Biese KJ, Busby-Whitehead J, Cai J (2018). Telephone follow-up for older adults discharged to home from the emergency department: a pragmatic randomized controlled trial. J Am Geriatr Soc.

[32] Shumway M, Boccellari A, O’Brien K (2008). Cost-effectiveness of clinical case management for ED frequent users: results of a randomized trial. Am J Emerg Med.

[33] Sadowski LS, Kee RA, VanderWeele TJ (2009). Effect of a housing and case management program on emergency department visits and hospitalizations among chronically ill homeless adults: a randomized trial. JAMA.

[34] Larimer ME, Malone DK, Garner MD (2009). Health care and public service use and costs before and after provision of housing for chronically homeless persons with severe alcohol problems. JAMA.

[35] California Department of Housing and Community Development (2024). Project homekey.

[36] Shinn GA (2014). The cost of long-term homelessness in Central Florida.

[37] Guleria I, Campbell JA, Thorgerson A (2025). Relationship between social risk factors and emergency department use: National Health Interview Survey 2016–2018. West J Emerg Med.

[38] National Advisory Committee on Rural Health and Human Services (2021). The future of rural health care.

[39] Di Mauro R, Di Silvio V, Bosco P (2019). Case management programs in emergency department to reduce frequent user visits: a systematic review. Acta Biomed.

[40] Kangovi S, Mitra N, Grande D (2020). Evidence-based community health worker program addresses unmet social needs and generates positive return on investment. Health Aff (Millwood).

[41] Noel L, Chen Q, Petruzzi LJ (2022). Interprofessional collaboration between social workers and community health workers to address health and mental health in the United States: a systematised review. Health Soc Care Community.

[42] Farmer P, Kim JY (2008). Surgery and global health: a view from beyond the OR. World J Surg.

[43] World Health Organization (2008). Task shifting: rational redistribution of tasks among health workforce teams: global recommendations and guidelines.

[44] Syed SB, Dadwal V, Martin G (2013). Reverse innovation in global health systems: towards global innovation flow. Global Health.

[45] Health Resources and Services Administration (2025). Rural health care services outreach program.

[46] Centers for Medicare & Medicaid Services (2026). Accountable health communities model.

[47] US General Accounting Office (2001). Emergency care: EMTALA implementation and enforcement issues.

[48] American College of Emergency Physicians (2013). EMTALA and on-call responsibility for emergency department patients. Policy statement.

[49] Woskie LR, Brower N, Shaffer J (2025). Obstetric-Related Emergency Medical Treatment and Labor Act violations and no health exception bans. JAMA Health Forum.

[50] American Nurses Association (2019). ANA health and safety survey: workplace violence and incivility.

[51] Kowalenko T, Walters BL, Khare RK (2005). Workplace violence: a survey of emergency physicians in the state of Michigan. Ann Emerg Med.

[52] US Bureau of Labor Statistics (2020). Workplace violence in healthcare, 2018 [fact sheet].

[53] Sharp AL, Cobb EM, Dresden SM (2014). Understanding the value of emergency care: a framework incorporating stakeholder perspectives. J Emerg Med.

[54] Shanafelt TD, Hasan O, Dyrbye LN (2015). Changes in burnout and satisfaction with work-life balance in physicians and the general US working population between 2011 and 2014. Mayo Clin Proc.

[55] Campbell EG, Regan S, Gruen RL (2007). Professionalism in medicine: results of a national survey of physicians. Ann Intern Med.

[56] Engel KG, Heisler M, Smith DM (2009). Patient comprehension of emergency department care and instructions: Are patients aware of when they do not understand?. Ann Emerg Med.

[57] Topol EJ (2019). High-performance medicine: the convergence of human and artificial intelligence. Nat Med.

[58] Obermeyer Z, Powers B, Vogeli C (2019). Dissecting racial bias in an algorithm used to manage the health of populations. Science.

[59] Wong A, Otles E, Donnelly JP (2021). External validation of a widely implemented proprietary sepsis prediction model in hospitalized patients. JAMA Intern Med.

[60] Wiens J, Saria S, Sendak M (2019). Do no harm: a roadmap for responsible machine learning for health care. Nat Med.

[61] Schneider SM, Gardner AF, Weiss LD (2010). The future of emergency medicine. J Emerg Med.

[62] Giwa A, Crutchfield D, Fletcher D (2021). Addressing moral injury in emergency medicine. J Emerg Med.

[63] Salhi RA, Macias-Konstantopoulos WL, Ryus CR (2023). Future directions to strengthen the emergency department safety net. West J Emerg Med.

[64] McClellan FM (2019). Healthcare and Human Dignity: Law Matters.

